# 1700 nm broadband laser source enables deep brain optical biopsy

**DOI:** 10.1038/s41377-021-00652-0

**Published:** 2021-10-04

**Authors:** Peijun Tang, Ruikang K. Wang

**Affiliations:** Department of Bioengineering, University of Washington, 3720 15th Ave NE, Seattle, WA 98195 USA

**Keywords:** Imaging and sensing, Biophotonics

## Abstract

An OCM system that employs a 1700 nm broadband laser source enables cellular level deep brain imaging, providing cytoarchitectural and myeloarchitectural information across cortical depth, without requiring tissue slicing. CC – corpus callosum.

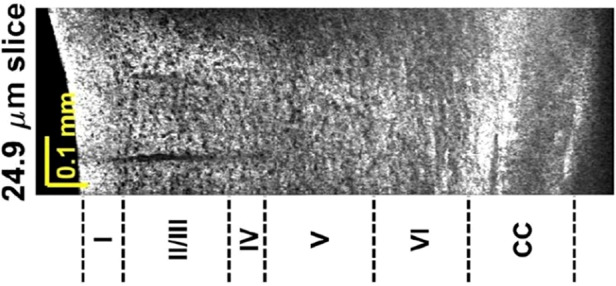

**A commentary on the article: Zhu et al. “1700 nm optical coherence microscopy enables minimally invasive, label-free, in vivo optical biopsy deep in the mouse brain.” Light: Science & Applications 10, 145 (2021).**

High-resolution optical imaging techniques are valuable as they can be used to provide brain tissue information at the cellular level in rodents. However, the intrinsic optical scattering and absorption properties of tissue often limit the imaging depth that these techniques can achieve. This limitation can be mitigated if the techniques are augmented with a number of additional considerations in the system design and/or implementation, for example, proper selection of a working wavelength that can minimize scattering and absorption effects and innovation in the experimental procedures that can facilitate easy access to the deep brain tissue, just to name a few. In this effort, researchers at the University of California Davis recently reported an optical microscopy system that employs a 1700 nm broadband laser source to achieve cellular-level deep brain imaging with minimally invasive procedures^1^. The team believes that such an imaging system would enable the development of new insights into neuropathology in the living, intact brain, such as in Alzheimer’s disease (AD) patients^2^.

The novel optical microscopy method demonstrated in paper^1^ is a type of imaging technique called optical coherence microscopy (OCM)^3^, which is an extension of the popular method of optical coherence tomography (OCT)^4,5^ but with much higher lateral resolution. We know that OCT utilizes a broadband light source to reconstruct three-dimensional images of turbid tissue with a typical spatial resolution between 10 and 30 µm. Since its advent, a number of useful OCT variants have been developed by leveraging other properties of light and tissue into the system design and development, enabling the implementation of additional contrast mechanisms for imaging the brain. These variants include OCT angiography^6,7^ to provide functional cerebral microvasculature^8,9^, polarization-sensitive OCT^10,11^ to provide information about nerve fiber tracts within the brain^12^, and phase-sensitive OCT to reveal neurovascular functions^13^. While these developments are important in investigations of brain functions, the lateral resolution cannot reach the single neuron level, leading to difficulty in deciphering several other important neural activities.

By combining the high-resolution optical microscopy technique (~1 μm) with OCT, OCM has been previously demonstrated to provide brain images at the individual neuron level^3,14^. Traditional OCM performed with a 1310 nm laser source requires invasive preparations in which overlying turbid tissues, such as the skull and dura, are removed to enable deep-brain imaging. However, invasive procedures significantly perturb brain physiology^15^. Compared with the 1310 nm wavelength, an optical window at 1700 nm results in significantly lower scattering while still enjoying overall relatively low water absorption. This property of a 1700 nm optical window can be harnessed to minimize ballistic attenuation and increase the imaging depth in the brain^16,17^. This advantage of a 1700 nm laser source was leveraged by this article^1^ to achieve cellular-level imaging in the deep cortical layers and beyond after a minimally invasive preparation.

The article reported that OCM coupled with a 1700 nm laser source can depict cellular details of cytoarchitecture and myeloarchitecture across the entire cortex in a rodent brain. Neuronal cell bodies, myelinated fibers, axons and plaques can be visualized from the mid-cortical to subcortical regions, more than 1 mm deep. Such imaging depth is critically important for neuropathological investigations related to central nervous system diseases that may occur deep in the brain, such as AD^2^. In the reported results, obvious differences between normal brains and those of AD mouse models were found in the deep layers, while such a difference was diminished within the superficial cortex, highlighting the importance of deep imaging in the AD model.

While this 1700 nm OCM system demonstrates its utility in deep brain imaging, there is an important caveat that should be recognized. That is, the depth of focus of the OCM is relatively short (~15 µm), leading to a narrow range of imaging depths that is in focus. Hence, to reconstruct an image in which all depths are in focus, several OCM volumes at different focal positions must be acquired and then digitally synthesized to form a final high-resolution volumetric OCM image. Even in a dynamic focus-tracking method (performed either manually or automatically), the imaging process requires a substantial increase in the time invested, causing nonnegligible time lags when different depths of the brain are targeted. This imitation would make the OCM technique extremely difficult to use to monitor the depth-dependent dynamic behaviors of the brain components, for example, the neuronal activity elicited by external stimulation.

Overall, the coherence gated technique of OCM provides imaging at greater depths than confocal-type microscopic technologies. The 1700 nm OCM approach achieves a unique balance between minimal invasiveness, resolution, and imaging depth for neuroimaging. The technical methodology demonstrated in this work will very likely be useful in many other deep brain imaging studies.
